# Microwave-assisted extraction enhances Aquafaba functionality: A high value-added egg white replacer in vegan meringue production

**DOI:** 10.1016/j.fochx.2025.102176

**Published:** 2025-01-13

**Authors:** Zahra Kargar, Abdollah Hematian Sourki

**Affiliations:** Department of Food Science and Technology, Faculty of Agriculture, Jahrom University, PO Box: 74135-111, Jahrom, Iran

**Keywords:** Aquafaba, Meringue, Microwave-assisted extraction, Thermogravimetric analysis, Turbidity, X-ray diffraction

## Abstract

This study aimed to compare microwave-assisted extraction (MAE) with conventional extraction methods regarding the physicochemical, techno-functional, molecular, and thermal properties of chickpea aquafaba. The potential of microwave-extracted aquafaba (MAEA) as an egg white replacer in vegan meringue production was also evaluated. The results indicated that while MAE reduced extraction yield and foam stability, it enhanced protein content, density, dry matter, and foaming ability compared to conventionally extracted aquafaba (CEA). X-ray diffraction revealed a reduction in protein crystal size, and FT-IR analysis confirmed the absence of harmful compounds in MAEA. Thermogravimetric analysis (TGA) identified key thermal degradation points. Substituting egg whites with aquafaba affected batter properties and meringue characteristics, but sensory evaluation showed no significant differences. Meringue with 50 % aquafaba substitution had the highest quality score, suggesting that this replacement offers economic and environmental benefits while meeting consumer preferences.

## Highlights

• Microwave-assistive process improved techno-functional properties of aquafaba.
• XRD showed microwave reduced aquafaba protein crystal size, changes C crystal to V.
• FT-IR revealed microwave extraction didn't produce harmful compounds in aquafaba.
• Replacing egg whites with aquafaba reduced batter viscosity of meringue.
• Sensory tests found no significant variance among egg white and aquafaba meringues.

## Graphical abstract

**Figure:**
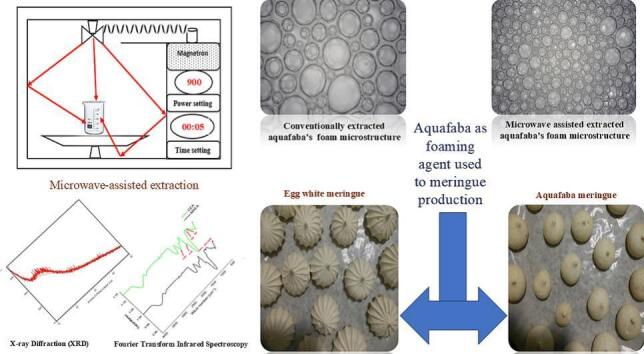

## 1 Introduction

The threats posed by climate change have driven researchers to modify the nutrition and dietary habits of consumers. Livestock farming directly contributes to 14.5 % of greenhouse gas (GHG) emissions (Gerber et al., 2013); however, considering the indirect sources of GHG emissions associated with livestock farming, this figure could be as high as 51 % (Goodland & Anhang, 2010). Moreover, livestock farming entails other negative impacts such as water scarcity, loss of biodiversity, animal suffering, antibiotic resistance, and shared diseases between humans and animals. By reducing or eliminating the consumption of animal products, some of these issues can be mitigated without compromising a healthy diet (González et al., 2011). A plant-based diet rich in protein sources such as legumes, grains, and nuts can fulfill these expectations (Aiking, 2014).

Legumes, such as chickpeas, beans, lentils, and soybeans, are rich sources of high-quality protein and can thus serve as significant substitutes for animal protein (He, Meda, et al., 2021). Additionally, they contain abundant carbohydrates that provide energy for human activity, metabolism, and growth. Legumes are also rich in dietary fiber and low in saturated fat. Among legumes, chickpeas have the best protein and carbohydrate quality, containing 20–26 % protein and 43–46 % starch (Alsalman & Ramaswamy, 2021a). However, some consumers are unwilling to forgo the taste, appearance, and performance of certain animal products. Therefore, researchers are striving to find alternative products that mimic the characteristics of meat, fish, dairy, and eggs (Shim et al., 2018). The replacement of eggs in food products poses a significant challenge. Apart from high nutritional value, the final product must simultaneously mimic several functions such as emulsifying, foaming, and gelling properties, while also effectively imitating color, taste and flavor (Yazici & Ozer, 2021).

Urbanization in recent decades has led to increased waste-release and exacerbated environmental damage (FAO, 2009). To address this issue, the efficient utilization of by-products, residues, and wastes generated by agricultural and food processing industries has emerged as a strategy to reduce waste and environmental hazards while creating high-value products. Aquafaba, the water obtained from cooking legumes, was previously considered waste but is now utilized as a highly nutritious plant-based material in food products (Stantiall et al., 2018). Aquafaba, with its unique functional properties, can be a suitable substitute for eggs in vegan products. Its composition, including 3–6 % protein, soluble carbohydrates, prebiotic compounds, soluble and insoluble fibers, and minerals, offers great potential for application in the food industry (He, Purdy, et al., 2021). Many functional properties of food products, such as foaming and emulsifying ability and stability, depend on their protein properties. Proper structure formation in pastries, water retention in bread and ice creams, reduction of oil absorption in doughnuts, and the formation and stabilization of fat emulsions in soups and cakes are among the most important functional properties of proteins (Shim et al., 2018).

The method of aquafaba extraction can have a significant impact on its yield and functional properties (Kargar & Hematian Sourki, 2024). Microwave-assisted extraction is a rapid and environmentally friendly method that is widely used in the food industry and for extracting bioactive compounds. Its rapid temperature increase, reduction in process time, space and energy savings, rapid penetration of heat into the center of the food material, and precise process control make it appealing to researchers and producers to use microwave technology in food processes (Ekezie et al., 2017). Microwave-assisted extraction of functional compounds from agricultural by-products has demonstrated significant advantages over conventional chemical extraction methods. For instance, Bedin et al. (2020) reported that MAE not only preserved the quality of rice bran protein but also achieved higher extraction yields and purity compared to alkaline extraction method. Similarly, Gonzalez-Rivera et al. (2023) demonstrated that the combination of microwave-assisted and ultrasonic extraction significantly enhanced the yield and qualitative properties of essential oils extracted from rosemary and lavender. As an eco-friendly extraction technique, MAE not only maintained the structural integrity of keratin but also enabled the direct production of highly electrospinnable nanofibers without requiring additional purification steps, offering substantial potential for bioplastic production (Pulidori et al., 2021).

Meringue is a product made from beating egg whites and sugar together, and is used to make pastries and cakes or to decorate them. Egg whites, due to their high foaming power, are one of the main components of this product and have a significant effect on its final quality. The relatively high cost of egg whites and their exclusion from vegan diets make plant-based ingredients with high foaming capabilities a promising alternative for these products. Studies have shown that limited researches have considered evaluating the effects of microwave on the properties of chickpea aquafaba and its application as an egg substitute in bakery products. Therefore, the objectives of this research are: A) To investigate the efficiency of microwave extraction on Kabuli chickpea (Adel variety) aquafaba and examine its physicochemical, molecular, thermal and techno-functional properties; B) To evaluate the potential substitution of egg whites with aquafaba and determine the physicochemical, techno-functional, and sensory properties of meringue containing aquafaba.

## 2 Materials and methods

### 2.1 Materials

Adel variety chickpeas were obtained from the Dryland Agricultural Research Institute of East Azerbaijan, Iran. Raw materials for the production of meringue included egg white, powdered sugar, citric acid, and vanilla, which were purchased from local supermarkets in the city of Jahrom, Iran.

### 2.2 Methods

#### 2.2.1 Microwave-assisted extraction of aquafaba

Pre-soaking, microwave application, and extraction of aquafaba were carried out using the optimized method described by Kargar and Hematian Sourki (2024). First, chickpea seeds were mixed with water at a ratio of 1:4 and soaked for 16 h. Then, the chickpea seeds were washed three times with tap water. In the conventional extraction method, approximately 100 g of soaked chickpea seeds were mixed with 300 mL of distilled water at a 1:3 ratio in a 500 mL glass beaker and were cooked at 120 °C in a pressure cooker (autoclave) for 20 min. Then, the cooked chickpeas and surrounded water were transferred to a glass container and kept in the refrigerator at 5 °C for 24 h. After 24 h, the water was separated from the cooked chickpeas using a sieve, and the separated water was used as aquafaba liquid for further experiments (Meurer et al., 2020).

In microwave-assisted extraction method, the soaked chickpea seeds (100 g) were mixed with 300 mL of distilled water and exposed to microwaves using a kitchen microwave oven (LG convection oven model SolarDOM). The microwave irradiation time were 5 min at 900 watts (Kargar & Hematian Sourki, 2024). After microwave treatment, the mixture of chickpea seeds and water was transferred to the pressure cooker (120 °C and 20 min). Then, the chickpea cooking process and aquafaba separation were performed according to the method mentioned above for the conventional extraction method. All aquafaba-related experiments were conducted on the same day of production.

#### 2.2.2 Physicochemical and techno-functional properties of aquafaba

Total protein content, pH, density, and dry matter content were determined using the Kjeldahl method, a pH meter (INSPECTED, PL-500 model), a pycnometer, and air oven drying method (AACC (2009)- 44–01), respectively (Fuentes Choya et al., 2023). For protein quantification, the aquafaba samples were initially frozen at −20 °C and then lyophilized using a freeze dryer (Alpha 2–4 LD Plus, Germany) at −30 °C and 13 Pa vacuum.

##### 2.2.2.1 Aquafaba extraction efficiency

The aquafaba extraction efficiency was calculated according to the method of Alsalman et al. (2020a), as the ratio of the weight of aquafaba to the weight of chickpeas consumed, expressed as grams of aquafaba per 100 g of chickpeas.

(1) $$\text{Extraction efficiency}\left(\%\right)=\frac{Q_{a}}{Q_{c}}\times 100$$

Where Qa was the weight of aquafaba (g) and Qc was the weight of chickpeas (g).

##### 2.2.2.2 Turbidity measurement

Turbidity of aquafaba was measured by determining the light transmittance of samples at a wavelength of 650 nm using a UV/VIS spectrophotometer (Jenway 7305 UV–vis spectrophotometer, England) and calculated using the following equation:

(2) $$\mathrm{Turbidity}=100*\left(T_{C}-T_{\mathrm{aq}}\right)∕\left(T_{C}\right)$$

Where T_C_ and T_aq_ represents the transmittance of deionized water and aquafaba respectively.

##### 2.2.2.3 Foaming ability (FA) and foam stability (FS)

Foaming ability and foam stability were determined according to the method described by Kargar and Hematian Sourki (2024). Thirty mL of aquafaba (V_A_) was poured into a 250 mL beaker and immediately mixed by an overhead stirrer (EUROSTAR 60, IKA Instruments, Germany) at 1500 rpm for 2 min to allow complete foaming. Then, the resulting foam was poured into a 250 graduated cylinder and the initial foam volume was read (V_i_). After 30 min, the foam volume was reread (V_30_). Foaming ability and foam stability were calculated via the following equations.

(3) $$\mathrm{FA}=\frac{V_{i}}{V_{A}}\times 100$$

(4) $$\mathrm{FS}=\frac{V_{30}}{V_{i}}\times 100$$

##### 2.2.2.4 Color properties of aquafaba foam

The color analysis of aquafaba foam samples was conducted using digital camera imaging (Casio) with the camera positioned 10 cm away from the sample under daylight conditions. Image processing was performed using ImageJ software (*v*ersion 1.52) to convert images to LAB color space, and parameters such as L* (lightness index), a* (green to red color spectrum), and b* (blue to yellow color spectrum) were calculated (Meurer et al., 2020).

##### 2.2.2.5 Microscopic characteristics of aquafaba foam bubbles

A light microscope (Model BX60, Olympus Optical Co, Ltd., Tokyo, Japan) with magnifications of 4 and 10 was used to investigate the structure of foam bubbles in aquafaba samples (Meurer et al., 2020). The bubbles pictures were captured by A digital camera (Leica EC3, Switzerland) that mounted on the microscope.

##### 2.2.2.6 Emulsifying capacity and stability

Emulsion capacity (EC) and stability were determined according to the method described by Alsalman and Ramaswamy (2021b). Oil-in-water emulsions (1:1 *v*/v) were prepared by mixing 15 mL corn oil with 15 mL aquafaba. The dispersions were pre-mixed using a magnetic stirrer for 2 min followed by homogenization with a laboratory overhead stirrer, EUROSTAR 60 (IKA Instruments, Germany) at 6000 rpm for 5 min at room temperature. The coarse emulsion was then sonicated for 7 min by using a 25 kHz ultrasonic processor (UP200Ht Hielscher, Germany) at a nominal maximum power output of 200 W and a cylindrical titanium sonotrode of 10 mm in diameter. Since the temperature increased during sonication process, a water-ice bath was used to maintain the temperature at about ambient. The emulsifying capacity was calculated using Eq. (5):

(5) $$E_{c}=\frac{V_{E}}{V_{T}}\times 100$$

where V_E_ is the emulsion volume and V_T_ is the total volume including serum and cream phase.

Emulsion stability (ES) against high temperature was determined by heating the emulsions in a water bath at 80 °C for 30 min followed by centrifuging at 4000 ×g for 10 min. ES was calculated using the following Eq. (6):

(6) $$\mathrm{ES}=\frac{V_{2}}{V_{1}}\times 100$$

where, V_1_ and V_2_ are the initial and final volume of emulsion respectively.

#### 2.2.3 X-ray diffraction (XRD)

XRD was used to determine the crystalline behavior, particle size, phases, and chemical composition of aquafaba proteins. The intensity of X-ray diffraction angles was analyzed using an X-ray diffractometer (CRYSTAL IMPACT, Bonn, Germany) with a scattering angle (2θ) ranging from 10 to 50 degrees and a step size of 0.01, with a wavelength of 1.541874 Å.

#### 2.2.4 Fourier transform infrared spectroscopy (FT-IR)

FT-IR analysis of lyophilized powdered aquafaba was performed by mixing it with potassium bromide and pressing it into tablet. The FT-IR analysis was carried out using a Bruker Tensor II FT-IR device equipped with an ATR accessory, in the wavenumber range of 400–4000 cm^−1^. A total of 32 scans at a resolution of 4 cm^−1^ used to observe infrared spectra.

#### 2.2.5 Thermogravimetric analysis (TGA)

Thermogravimetric analysis of lyophilized powdered aquafaba was conducted using a TGA-DSC thermogravimetric device (Mettler Toledo, Switzerland) under nitrogen gas pressure. The weight changes of 5 mg of lyophilized aquafaba powder under temperature changes were recorded from 25 to 600 °C at a rate of 10 °C/min.

#### 2.2.6 Production of vegan meringue using aquafaba

Vegan meringue and control sample was produced by mixing 50 g of foaming agent (according to the treatment schedule in Table 1) with a laboratory mixer for 3 min until it became light and fluffy. During mixing, 70 g of powdered sugar was gradually added, and mixing continued until the mixture became slightly firm and formed. Then, 0.15 g of citric acid (pH adjuster) and 5 g of vanilla were added and mixed. The mixture was placed on baking trays covered with silicone baking mats and then baked in a preheated oven at 100 °C for 20 min. The prepared meringues were cooled completely after 30 min and stored in polyethylene bags for further analysis (Yüceer & Caner, 2021). The meringue containing egg white was referred to as the control sample, while the recipes with 25 %, 50 %, 75 %, and 100 % substitution of egg white with MAEA were designated as Aqu25, Aqu50, Aqu75, and Aqu100, respectively (Table 1).

**Table 1:** Meringue recipes with varying percentages of egg white replacement by aquafaba.

**Ingredients (g)**	**Control**	**Aqu25**	**Aqu50**	**Aqu75**	**Aqu100**
Egg white	50	37.5	25	12.5	0
Aquafaba	0	12.5	25	37.5	50
Powdered sugar	70	70	70	70	70
Citric acid	0.15	0.15	0.15	0.15	0.15
*V*anilla	5	5	5	5	5

#### 2.2.7 Physicochemical properties of vegan meringue

The viscosity and flow behavior of meringue batter were measured using a rotary viscometer (model NDJ—8S) at shear rate 100 S^−1^. The specific density and pH of meringue batter, specific volume, and color analysis of meringue were measured using weighing, a pH meter (INSPECTED, PL-500 model), the rapeseed displacement method (AACC (2009) standard number 50–10), and image processing with Image J software (version 1.52), respectively (Yüceer & Caner, 2021).

#### 2.2.8 Sensory evaluation of vegan meringue

Ten graduate students majoring in Food Science and Technology at Jahrom University underwent preliminary training on sensory evaluation and were screened and selected. The five-point Hedonic scale (1 equivalent to the minimum score and 5 equivalent to the maximum score) was used for sensory evaluation. The appearance, taste, flavor, texture (uniformity and firmness), color, and overall acceptance were evaluated.

#### 2.2.9 Experimental design and statistical analysis

The *t*-test was used for comparing the physicochemical properties of MAEA with CEA sample. All experiments were conducted in triplicate. Analysis of variance was performed using JMP software (version 8.0). Mean values were compared using Duncan's multiple range test at a significance level of 95 %.

## 3 Results and discussion

### 3.1 Physicochemical and techno-functional properties of aquafaba

#### 3.1.1 Extraction efficiency

The extraction efficiency for the MAEA was 175.53 %, while for the CEA it was 185.51 % (Table 2). With the application of microwave treatment and the consequent evaporation of water used for aquafaba extraction, the extraction efficiency decreased compared to the CEA. Microwaves are essentially non-ionizing radiation energy. Due to their periodic interaction, they cause molecular friction, resulting in instantaneous heat generation in water molecules (Divekar et al., 2017). Marconi et al. (2000) reported that the yield of cooked chickpea water (aquafaba) decreased with microwave treatment compared to the conventional method (no microwave treatment). They believed that this decrease was due to the microwave treatment at high temperature and pressure, resulting in the evaporation of a large volume of consumed water, which led to a reduction in extraction efficiency.

**Table 2:** Physicochemical properties of microwave-assisted extracted aquafaba compared to conventionally extracted aquafaba and egg white.

Physicochemical properties	MAEA	CEA	Egg white
Extraction efficiency (%)	175.53 ± 1.12	185.51 ± 1.62	–
Total protein (%)	22.61 ± 0.79	20.26 ± 0.57	–
Density (g/mL)	1.04 ± 0.03	1.02 ± 0.03	1.06 ± 0.04
Dry matter (%)	4.97 ± 0.11	4.60 ± 0.08	13.63 ± 0.31
Foaming ability (%)	276.66 ± 1.58	326.66 ± 1.33	256.66 ± 2.01
Foam Stability (%)	50.00 ± 0.89	41.37 ± 0.77	45.00 ± 0.62
Emulsifying Capacity (%)	60.00 ± 0.88	50.00 ± 0.94	–
Emulsion Stability (%)	26.66 ± 0.32	7.69 ± 0.08	–
Turbidity (%)	95.67 ± 1.05	91.53 ± 0.68	9.00 ± 0.03
pH	6.56 ± 0.21	6.57 ± 0.13	8.97 ± 0.09
Lightness (L*)	75.77 ± 3.14	72.02 ± 2.68	70.45 ± 2.60
Redness (a*)	- 0.28 ± 0.09	- 0.74 ± 0.08	- 1.61 ± 0.07
Yellowness (b*)	- 4.80 ± 0.19	- 4.50 ± 0.22	- 1.44 ± 0.56

All data were expressed as mean ± SD of three replicates.

#### 3.1.2 Total protein

The results showed that the total protein content of MAEA was 22.61 % (dry weight basis-DWB), while the protein content of the CEA was 20.26 % (Table 2). Statistical analysis showed a significant difference between the effect of extraction method on the total protein content (*p* < 0.05). Solé Lamich (2022) reported that approximately 18 to 29 % of the dry matter of chickpea aquafaba consists of proteins. Marconi et al. (2000) reported that the MAE led to a decrease in the protein content of cooked chickpea water. They first precooked the chickpeas in one stage and after draining and removing the cooking water, the chickpeas were subjected to microwave treatment, followed by complete cooking. Since they separated the precooking water from the chickpeas once, a significant portion of the aquafaba protein may have been discarded with the discarded precooking liquid, resulting in a reduction in the total protein content of the aquafaba compared to the control sample.

#### 3.1.3 Density

The analysis of variance results showed no significant difference between the density of MAEA (1.04 ± 0.03 g/mL) and CEA (1.02 ± 0.03 g/mL) (*p* > 0.05) (Table 2). However, the density of aquafaba was slightly lower than that of egg white (1.06 ± 0.04 g/mL). Tufaro and Cappa (2023) reported the density of chickpea aquafaba as 1.01 ± 0.02 g/mL. The reason for the higher density of MAEA compared to the CEA is likely the thermal energy effect of microwaves on cooking and disrupting the structure of chickpeas, facilitating the release of more soluble solid components, including proteins, carbohydrates, and minerals during cooking and thermal processes, which increases the density. Divekar et al. (2017) reported that analysis of scanning electron microscope (SEM) images of chickpeas and lentils after microwave treatment showed the creation of cracks in the seed coat and cotyledons. These cracks can facilitate the release of soluble compounds, including proteins, carbohydrates, and minerals, during cooking and thermal processes.

#### 3.1.4 Dry matter

The results showed that the dry matter content of MAEA and CEA was 4.97 and 4.6 % (wet weight basis-WWB), respectively, which differed significantly from each other (*p* < 0.05) (Table 2). The reason for the increase in the dry matter of MAEA is the further destruction of the chickpea tissue compared to the CEA, which leads to the release of more soluble solid compounds into the cooking water and increases the dry matter content of the MAEA compared to the CEA. Additionally, the results showed that the dry matter content of aquafaba was much lower than that of egg white (13.63 %), indicating the high economic value of this product. Aquafaba dry matter contains soluble and insoluble carbohydrates, ash, insoluble fiber, protein, and saponins, which exhibit different technological properties. With increasing duration and intensity of microwaves, the detrimental effect of their heating on chickpea tissue increases, and the original integrity is lost, leading to increased permeation of solvent and release of soluble solid compounds such as carbohydrates, proteins, and minerals from inside the chickpea tissue to the solvent, resulting in increased dry matter of aquafaba (Ates et al., 2024).

#### 3.1.5 Foaming ability

The foaming ability of MAEA, CEA, and egg white was calculated as 276.66 %, 326.66 %, and 256.66 %, respectively (Table 2). The results indicated that the foaming ability of Kabuli chickpea aquafaba was significantly higher than that of egg white, which slightly decreased with microwave heat treatment during extraction, but still remained higher than egg white. Lafarga et al. (2019) reported the foaming ability for chickpea aquafaba as 162–324 %. Additionally, Meurer et al. (2020) reported the foaming ability for chickpea aquafaba as 259 %.

Aquafaba contains water-soluble carbohydrates, insoluble fiber, proteins, and saponins, each exhibiting different technological properties (Bird et al., 2017; Stantiall et al., 2018). Suhag et al. (2021) reported that microwave radiation intensity and duration significantly affect the inactivation of non-nutritional compounds (such as phytic acid, tannins, saponins, and oxalates) in many cereal grains, attributed to microwave-induced sensitivity due to thermal degradation resulting in insoluble complex formation. Saponins in aquafaba enhance foaming properties by aggregating at the air/water interface, reducing interfacial tension, and aiding in foam formation (Bird et al., 2017; Stantiall et al., 2018). Therefore, increasing microwave time and power led to the decomposition of some saponins, reducing aquafaba's foaming ability. Conversely, intense microwave heating can denature soluble proteins, converting them into insoluble forms, potentially reducing the foaming ability of Kabuli chickpea aquafaba (Ates et al., 2024).

#### 3.1.6 Foam stability

The foam stability of MAEA was calculated as 50 %. The foam stability of CEA was 41.37 %, and for egg white, it was 45 % (Table 2). Meurer et al. (2020) reported that the foam stability for chickpea aquafaba as 77–42 %. Lafarga et al. (2019) reported foam stability for aquafaba as 36.9–93.4 %. They reported that aquafaba proteins accumulate at the air-water interface, reducing surface tension and partially unfolding proteins, allowing fewer air bubbles to be trapped and enabling protein molecules to stabilize the foams. They also mentioned that aquafaba polysaccharides, due to their hydrophilic nature and high molecular weight, possess water-holding and thickening properties that can increase foam stability by gelation or altering the viscosity of the aqueous phase, thereby enhancing the stability of aquafaba foams.

#### 3.1.7 pH

The pH of MAEA, CEA, and egg white was measured as 6.56, 6.57, and 8.97, respectively (Table 2). There was no significant difference in the pH of aquafaba samples, indicating that the extraction method had no significant impact on their pHs (*P* > 0.05). Similarly, other studies have reported that aquafaba's pH, regardless of the extraction method, generally falls within the range of 6 to 7. In this regard. Tufaro and Cappa (2023) reported that the pH values for aquafaba and egg white were 6.15 and 8.41, respectively.

#### 3.1.8 Turbidity

The turbidity of MAEA, CEA, and egg white was 95.67 %, 91.53 %, and 9 %, respectively (Table 2). Wang et al. (2018) reported that protein dispersion and aggregation increase turbidity. Mohan et al. (2008) stated that turbidity measurement directly reflects the scattering status, aggregation state, and particle size of proteins in solution. In a study by He et al. (2022), it was shown that the turbidity of quinoa extract gradually increased with time and temperature. Cromwell Cromwell et al. (2006) found that increased turbidity accompanies the formation of protein aggregates. Increased turbidity in MAEA is due to the higher temperature created by microwave treatment, increasing intramolecular and intermolecular protein interactions, resulting in significant protein aggregation and increased turbidity (Li et al., 2013). Additionally, as mentioned in the dry matter section, the application of microwave treatment increased the dry matter content of the aquafaba solution, likely due to the entry of more polysaccharides, proteins, and minerals into aquafaba, which can increase its turbidity.

#### 3.1.9 Emulsifying capacity

The emulsifying capacity of MAEA was 60 %, while it was 50 % for the CEA (Table 2). Lafarga et al. (2019) reported the emulsifying capacity for chickpea aquafaba as 3.9–72.3 %. Solé Lamich (2022) reported that the emulsifying capacity of aquafaba ranged from 54.2 % to 68.5 %. Alsalman et al. (2020a) reported that aquafaba with longer cooking times had higher emulsifying capacities. Since microwave treatment provides longer cooking time compared to the conventional method, the emulsifying capacity of MAEA was higher than the CEA. They also reported that longer cooking times denature more chickpea proteins, leading to increased emulsifying properties of aquafaba.

#### 3.1.10 Emulsion stability

The emulsion stability of MAEA and CEA was 26.66 % and 7.69 %, respectively, showing a significant difference (Table 2). Lafarga et al. (2019) reported emulsion stability for aquafaba as 0–76 %, and He, Purdy, et al. (2021) reported emulsion stability for aquafaba as 72–76 %. Mustafa Mustafa et al. (2018) reported that the polysaccharides present in aquafaba increase emulsion stability by increasing the viscosity of the aqueous phase. The dry matter content (5–8 %) and protein content (0.85–1.5 % w.b) in aquafaba are other factors increasing emulsion stability. Generally, more stable emulsions, such as protein-oil-water emulsions, contain low protein concentrations (0.2–1 %). In this regard, Alsalman et al. (2020a) reported that aquafaba with longer cooking times had higher emulsion stability. Since microwave treatment provides longer cooking time compared to the conventional method, the emulsion stability of MAEA was higher than the CEA. The denaturation of proteins by microwave irradiation by Bohr and Bohr (2000) and de Pomerai et al. (2003) have also been reported.

#### 3.1.11 Color properties of aquafaba

The results showed that in MAEA, the b* value decreased compared to the CEA, while the values of a* and L* increased (Table 2). Tufaro and Cappa (2023) reported that the L* value of the aquafaba sample did not have a significant difference compared to egg white, and the a* value of aquafaba was higher than egg white, while the b* value of aquafaba was lower than egg white, which was consistent with our results. The dark appearance and yellowish color of aquafaba mainly derive from pigment residues of seed coat. Wetting and thermal processing eliminate these pigments, resulting in a lighter appearance (L*), less yellowness (b*), and less color difference. Considering that microwave treatment involves a higher level of thermal processing, it leads to an increase in the lightness (L*) of aquafaba (Xu et al., 2017).

#### 3.1.12 Microscopic visualization of aquafaba foam structure

**Fig. 1:**
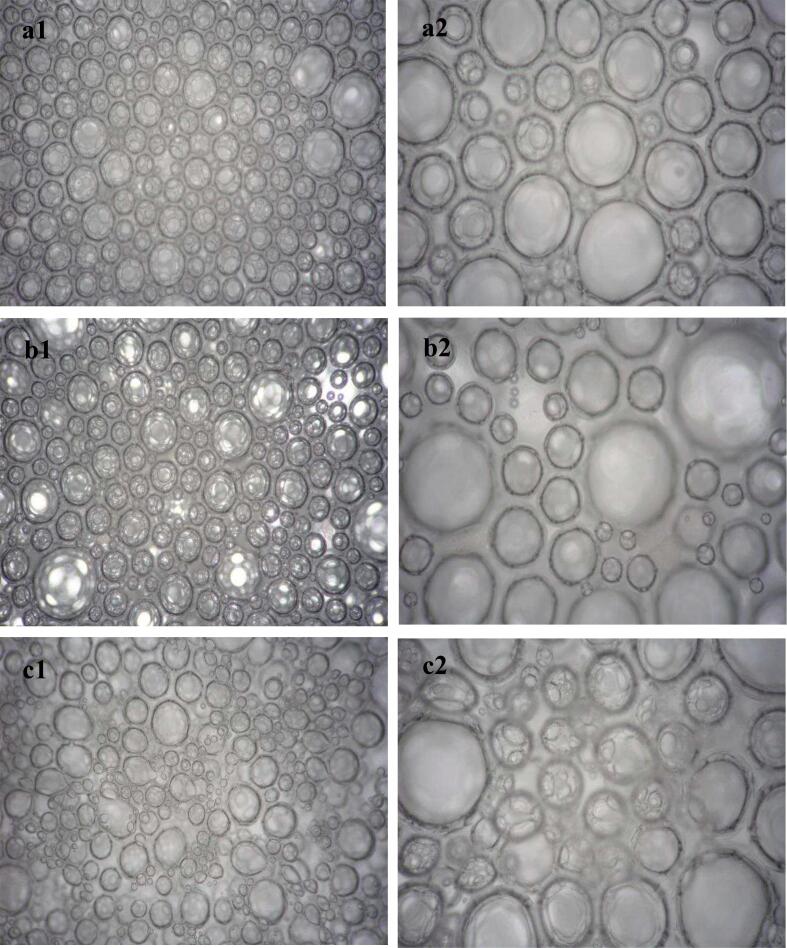
Microscopic visualization of foaming agents. a1: MAEA with 4× magnification; a2: MAEA with 10× magnification; b1: CEA with 4× magnification; b2: CEA with 10× magnification; c1: egg white with 4× magnification; c2: egg white with 10× magnification.

Microscopic examination of the aquafaba foam bubbles showed that the MAEA had a similar bubble diameter and uniform distribution of bubble size to egg white foam, while the CEA had larger bubbles with a less uniform size distribution (Fig. 1). Bennion et al. (1997) believed that smaller and uniform bubbles contribute to stability and provide a good texture for food. Changes in bubble size and the formation of larger bubbles create an unstable mixture and a rough texture for the product.

### 3.2 X-ray diffraction (XRD)

**Fig. 2:**
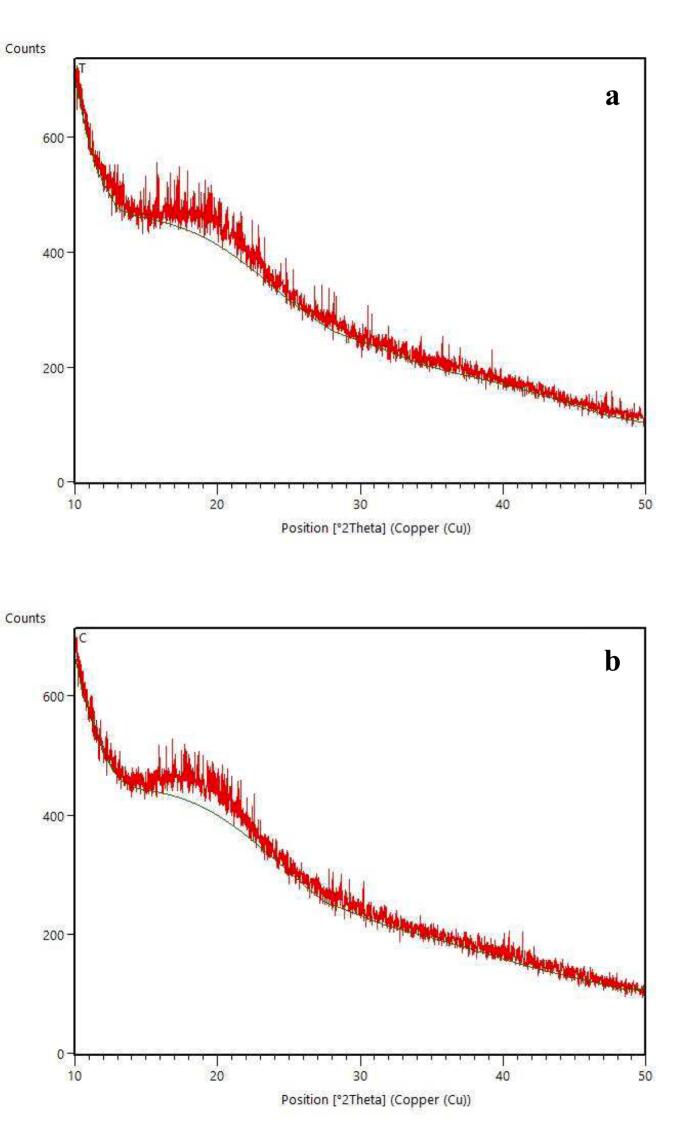
XRD patterns of microwave-assistive extracted aquafaba (a) and conventionally extracted aquafaba (b).

According to Fig. 2, peaks can be observed at approximately angles of 15, 16, 17, 18, 20° for the control aquafaba sample, which corresponds to the crystallinity of type C. While in Fig. 2, for the MAEA, peaks with higher intensity appeared in the range of angles 17–20°, which is due to the thermal effect of microwaves and the transformation of crystallinity from type C to type *V*.

Alsalman et al. (2020b) reported that the crystallinity type of aquafaba may be type V, in which the main scattering peak is at 20°. Xu et al. (2019) found that autoclaving changes the crystallinity of chickpeas from type C to type V, which was caused by the heat generated by microwaves in our study.

The results showed that the scattering intensity of the CEA was relatively higher than that of the MAEA, which is probably because microwaves increase the heat, leading to protein denaturation and consequently the opening of its structure. Thermal processing leads to a decrease in the crystallinity percentage of the MAEA compared to the CEA (Fig. 2). In line with these results, Mir et al. (2021) and Malik and Saini (2018) reported that the crystal size decreases after thermal processing, which can be attributed to the denaturation of protein molecules and the subsequent interaction of denatured molecules. The interaction of denatured protein molecules leads to the formation of new chemical bonds that can disrupt the regular arrangement of protein molecules, reduce overall strength, and thus reduce crystal size.

### 3.3 Fourier transform infrared spectroscopy (FT-IR)

**Fig. 3:**
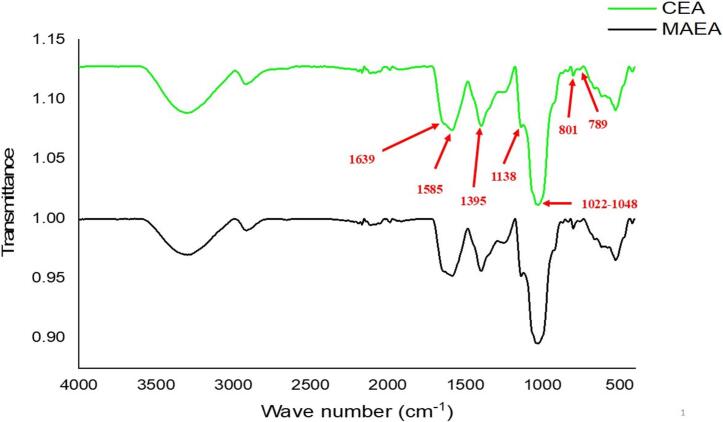
FT-IR spectra of microwave-assistive extracted aquafaba (MAEA) and conventionally extracted aquafaba (CEA).

The FT-IR analysis showed sharp absorption peaks at wave numbers of 801.33, 1028.45, and 1138.44 cm^−1^, which are probably related to the presence of carbohydrates in the aquafaba composition (Fig. 3). Rozenberg et al. (2019) reported that in FT-IR spectroscopy, the carbohydrate spectrum ranged 800–1300 cm^−1^, which is also called the fingerprint region.

Divekar et al. (2017) reported that proteins contain C=O, C—N, and N—H bonds. The vibration of amide I is primarily due to C=O stretching (80 %) and C—N stretching vibrations and has a characteristic peak at 1600–1700 cm^−1^. Alsalman and Ramaswamy (2021b) suggest that peaks in the range of 1600–1700 cm^−1^ correspond to amide I, indicating the secondary structure of proteins. As shown in Fig. 3, the peak at 1639 cm^−1^ confirms the presence of proteins in both CEA and MAEA samples. Amide type II has absorption peaks at 1500–1600 cm^−1^, which indicates N—H bending vibrations (60 %) and C—N stretching vibrations (40 %). Accordingly, the absorption peak observed at 1585.53 cm^−1^ is probably related to the presence of proteins in aquafaba composition.

Starch crystallinity can be measured by FT-IR through an absorption ratio at 1022–1048 cm^−1^, where the absorption at 1048 cm^−1^ indicates the crystalline region and molecular arrangement, and absorption at 1022 cm^−1^ related to the amorphous region and amorphous granules of starch (Pozo et al., 2018; Xu et al., 2019). The results showed an intense absorption peak at 1028.45 cm^−1^ wave number, which is probably related to starch (Fig. 3). Xu et al. (2019) reported that in FT-IR spectroscopy of chickpeas, absorption peaks at 1022–1048 cm^−1^ related to starch. Pozo et al. (2018) reported that corn, wheat, and chickpea starch had an absorption peak at 1022–1048 cm^−1^. The absorption peak at 801.33 cm^−1^ was probably related to amorphous regions in aquafaba starch content (Fig. 3). In this regard, Kamble et al. (2020) reported that the absorption peak at 789.52 cm^−1^ was attributable to the C-O-H group, thus describing the amorphous state in starch (Kamble et al., 2020).

The results of FT-IR spectroscopy showed no significant difference between the spectrum of the CEA and MAEA. Almost all major peaks were observed without much or significant shift in both spectra. A lack of differences between the FT-IR spectrum of the CEA and MAEA indicated that microwave irradiation has negligible effects on the chemical structure of compounds in aquafaba. It was observed that microwave irradiation did not lead to the production of unwanted compounds in aquafaba.

### 3.4 TGA analysis

**Fig. 4:**
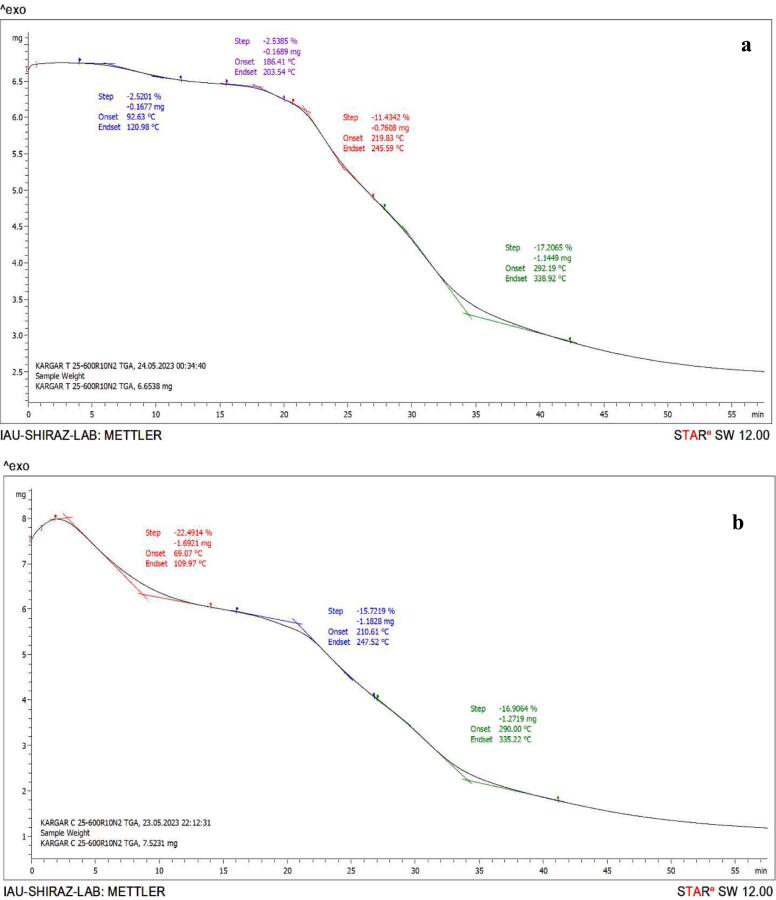
TGA curves of microwave-assistive extracted aquafaba (a) and conventionally extracted aquafaba (b).

TGA shows relationships between loss of mass and temperature changes during an experiment. It helps to evaluate the basic mechanism for degradation as well as the reaction kinetics. TGA graph showed that the first peak of weight loss in both samples occurred by the evaporation of free and bound water (Fig. 4) (Chawla et al., 2023). Also, Lamo et al. (2022) reported that the initial weight loss in temperature ranges from 30 to 150 °C may be attributed to the evaporation of water molecules in samples. The first peak in the TGA graph of the CEA was more intense and occurred at a lower temperature, which can be due to a lower protein accumulation and lower fat content of the CEA, compared to the MAEA, hence its higher water binding capacity. However, the optimal sample had lost some of its moisture during microwave treatment due to heat treatment.

The second peak in the TGA graph of MAEA was in temperature ranges of 186–203 °C, which is probably related to fat degradation, although not apparent in the graph of the CEA (Fig. 4). Huntrakul et al. (2020) reported that an additional peak appeared at 193.75–197.75 °C in solutions containing stearic acid, which can be attributed to the evaporation of fatty acids.

The third peak of the MAEA was observed in the TGA graph at 219–245 °C and the second peak in the TGA graph of CEA at 210–247 °C (Fig. 4). Weight loss at 200–450 °C was attributed to the thermal degradation of proteins due to subsequent evaporations caused by breakage in covalent bonds in existing amino acids and disulfide bonds (Singh & Sogi, 2018). At temperatures above 100 °C, proteins decompose into their subunits and support the formation of protein aggregates through hydrophobic, electrostatic, and disulfide bonding mechanisms. Ricci et al. (2018) stated that a weight loss step in the temperature range of 178–288 °C in the TGA curve for chickpeas and other legumes is attributed to the volatilization of protein fragments generated by thermal-activated reactions (decomposition). This temperature range may vary depending on the nature and purity of the proteins. Sharma and Singh (2016) indicated that weight loss in sesame protein film at 220 °C occurred due to the evaporation of glycerol and the splitting of covalent bonds among amino acid groups in proteins. The last peaks pertaining to the MAEA at 292–338 °C and for the CEA at 290–335 °C were attributed to the complete breakdown of proteins (Zhang et al., 2019).

### 3.5 Physicochemical and sensory properties of meringue

#### 3.5.1 Bulk Specific Density (BSD) of Meringue batter

The results indicated that as the proportion of aquafaba increased from 0 to 100 %, as a replacement of the egg white, the bulk density of meringue batter increased from 0.3 to 0.49 g/mL (Table 3). The ability of aquafaba to produce meringue was relatively lower than that of egg white. Therefore, with the increase in the substitution of egg white with aquafaba, the ingress of air into the batter decreased, leading to an increase in the density of the meringue batter. On the other hand, due to the lower viscosity of aquafaba compared to egg white, the meringue batter containing aquafaba appeared more diluted. This issue caused instability in the foam bubbles inside the batter, leading to some air escaping from the batter. Yüceer and Caner (2021) reported that the density of the batter is related to the volume fraction of air. They also reported that adding protease to the meringue batter reduced the bulk density, which may be due to the deleterious effect of protease on albumin proteins and the decrease in its ability to produce foam. Similarly, Wouters et al. (2018) obtained bulk density of meringue batter between 0.3 and 0.5 g/mL.

**Table 3:** The physicochemical, and color properties of meringue batter containing varying amounts of MAEA.

Meringue batter recipes	Bulk Specific Density (g/mL)	pH	Lightness (L*)	Redness (a*)	Yellowness (b*)
Aqu100	0.49 ± 0.01	6.00 ± 0.12	66.97 ± 1.32	- 0.08 ± 0.01	5.49 ± 0.08
Aqu75	0.34 ± 0.02	7.01 ± 0.10	66.61 ± 4.22	- 0.20 ± 0.01	4.95 ± 1.02
Aqu50	0.30 ± 0.01	7.08 ± 0.15	64.34 ± 4.39	- 0.86 ± 0.01	5.52 ± 0.06
Aqu25	0.32 ± 0.02	7.39 ± 0.09	67.99 ± 2.87	- 0.61 ± 0.01	5.16 ± 0.04
Control	0.36 ± 0.02	7.30 ± 0.15	68.30 ± 2.77	- 0.75 ± 0.01	5.01 ± 0.02

All results were expressed as mean ± SD of three replicates.

#### 3.5.2 pH of meringue batter

The results showed that with the increase the ratio of aquafaba in the meringue batter, the pH of the samples decreased (Table 3). In fact, given the alkaline pH of egg white, as the proportion of aquafaba increased and consequently the proportion of egg white decreased, the pH of the batter decreased. The pH of chickpeas aquafaba was 6.56, while the pH of egg white was 8.97.

#### 3.5.3 Viscosity of meringue batter

**Fig. 5:**
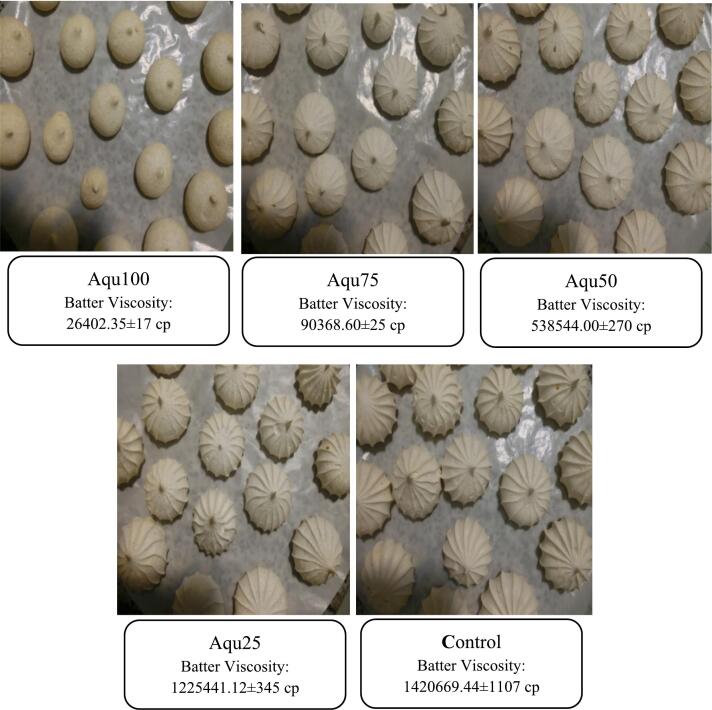
The relationship between the viscosity of meringue batter and the appearance of meringues influenced by different levels of egg white replacement with MAEA.

The viscosity of the meringue batter decreased significantly (*p* < 0.05) with the increase in aquafaba ratio than egg white (Fig. 5). Egg white, due to the presence of proteins with thickening properties such as ovomucoid and ovomucin, has a higher viscosity compared to aquafaba. Alsalman et al. (2020b) stated that aquafaba that had a longer cooking time and lower water content had a higher viscosity. The reduction in the viscosity of meringue batter containing aquafaba resulted in the loss of the initial shape of the meringue after baking (Fig. 5). In fact, it seems that the main factor affecting the changes in the density of the batter and the bulk specific volume of the meringue was the lower viscosity of aquafaba compared to egg white, which also had an adverse effect on the appearance of the Aqu75 and Aqu100 meringue samples (Fig. 5).

#### 3.5.4 Evaluation of meringue batter Color

The results showed that with the increase in the ratio of aquafaba in the meringue batter, the values of b* and a* increased, while L* decreased significantly (Table 3). The release of yellow carotenoid pigments from the chickpea skins and their entry into aquafaba may be the cause of the increased b* (yellowness) of the meringue batter (He et al., 2019). Aquafaba contains significant amounts of proteins and soluble carbohydrates, which easily participate in Maillard browning reactions, resulting in darker color and decreased L* of the meringue batter. Manisha et al. (2012) believe that changes in the batter density through air ingress into the batter can also affect the color of the batter.

#### 3.5.5 Bulk specific volume (BSV) of meringue

The results showed that with the increase in the proportion of egg white in the meringue instead of aquafaba, the bulk specific volume of the meringue increased. The reason for this was the higher ability of egg white to produce foamy meringue and the greater stability of it during the baking process (Table 4) (Yüceer, 2020). Yüceer (2020) attributed the increase in bulk specific volume of meringue samples to the decrease in the density of the meringue batter. The increase in the bulk specific volume of baked meringue is related to the absorption of air and the ability to retain the gas during the mixing process. Moreover, the bulk specific volume of meringue can usually be controlled by using a mixer, changing the speed of rotation, the temperature of the batter, and the type of raw materials. Meringues with higher bulk specific volume showed the least hardness, which may be related to the size of the air bubbles in the foam matrix (Yüceer & Caner, 2021).

**Table 4:** The physicochemical, and color properties of meringue containing varying amounts of MAEA.

Meringue recipes	Bulk Specific Volume (mL/g)	Lightness (L*)	Redness (a*)	Yellowness (b*)
Aqu100	14.53 ± 0.25	70.13 ± 2.10	- 2.85 ± 0.76	16.66 ± 2.34
Aqu75	16.49 ± 0.22	71.03 ± 5.00	- 1.34 ± 0.79	10.08 ± 1.47
Aqu50	17.51 ± 0.37	73.02 ± 4.16	- 1.98 ± 0.88	13.01 ± 2.11
Aqu25	19.54 ± 0.31	63.62 ± 3.70	- 1.10 ± 0.82	13.52 ± 1.50
Control	22.03 ± 0.60	68.99 ± 3.65	- 0.71 ± 0.89	13.19 ± 1.64

All data were expressed as mean ± SD of three replicates.

#### 3.5.6 Evaluation of baked meringue color

The results showed that with the increase in the ratio of aquafaba, the b* value of the meringue increased, while the L* and a* indices decreased significantly (p < 0.05) (Table 4). For a* values (greenness), there was a significant difference between the control and Aqu25 samples compared to the Aqu100 sample (p < 0.05). And also, for b* values, there was a significant difference between the control sample and the Aqu50, Aqu75, and Aqu100 samples (p < 0.05). The highest lightness (L*) belonged to the Aqu50, while the lowest belonged to the Aqu25. The highest b* value belonged to the Aqu100, while the lowest belonged to the Aqu75. The control sample and the Aqu25 had the highest and lowest a* values, respectively. Fuentes Choya et al. (2023) reported that the presence of large amounts of protein in aquafaba along with large amounts of sugar in meringues recipe and applying a temperature of 90 °C for a long-time during cooking increased the hydrolysis of sucrose glycosidic bonds, which increased the reducing power of sucrose and thus increased the rate of the Maillard reaction. An increase in Maillard reactions makes the color darker and reduces the L*of the meringue color.

#### 3.5.7 Sensory analysis of meringue

**Fig. 6:**
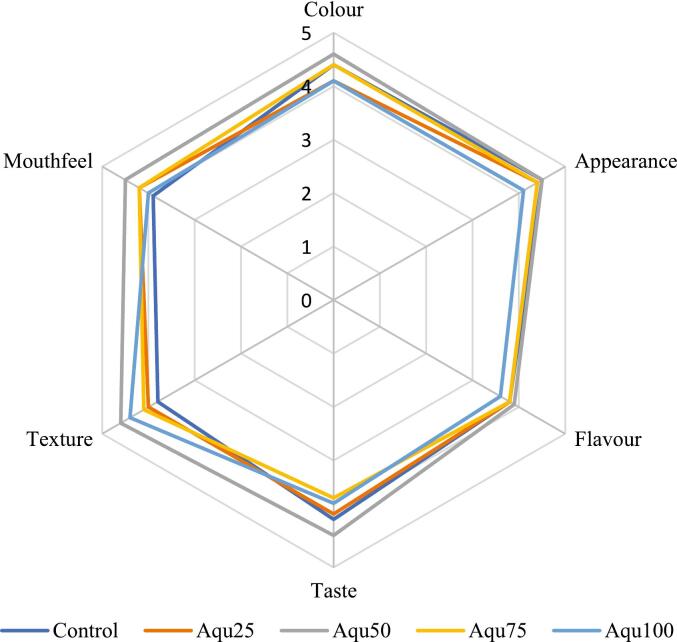
Sensory evaluation radar chart of meringue containing varying amounts of MAEA.

Statistical analysis of sensory characteristics of meringue, including color, appearance, aroma, taste, texture, and mouthfeel, showed that none of these sensory features differed significantly among samples containing different ratios of chickpea aquafaba compared to the control sample (*p* > 0.05) (Fig. 6). The Aqu50 scored the highest in all sensory attributes of meringue, indicating that aquafaba not only has no detrimental effect on the quality of meringue but also has equal or even better acceptability and palatability than egg whites.

Lafarga et al. (2019) reported that in the sensory evaluation of meringue containing aquafaba, overall acceptance increased compared to the control sample, and no differences were observed in taste, texture, and color, with only a slight reduction in texture firmness. In line with this, Fuentes Choya et al. (2023) stated that meringues containing aquafaba received high scores and were similar to those containing egg whites.

## 4 Conclusion

Due to its low-cost and high production capacity, aquafaba can be a suitable, low-cost, and valuable option for replacing egg whites in meringues and other vegan products. In general, the results showed that the use of microwave radiation in aquafaba extraction, although it reduced the extraction efficiency and the ability to produce aquafaba foam, had a beneficial effect on its physicochemical properties, including total protein content, density, dry matter, foam stability, emulsifying capacity, emulsion stability, and turbidity compared to CEA sample. Substituting egg whites with MAEA in meringue batter resulted in increased bulk density, improved bulk specific volume, and reduced viscosity of the batter, which could facilitate the production process of this type of confectionery. In general, the sensory evaluation showed that there was no significant difference between the vegan meringue containing aquafaba and the meringue containing egg white, and the panelists gave the highest quality score to the Aqu50. Considering the lack of change in appearance of Aqu50 meringue compared to the control sample, this formulation can be suggested as the best and most economical formulation of meringue. It seems that using at least 50 % of aquafaba instead of egg white in meringue will reduce the environmental damage caused by wastewater disposal and increase the economic efficiency of canning industries.

## Ethical statement

This study did not include any human subjects and animal experiments.

## Consent for publication.

All authors listed have read the complete manuscript and have approved submission of the paper.

## Funding statement

This research did not receive any specific grant from funding agencies in the public, commercial, or not-for-profit sectors.

## CRediT authorship contribution statement

**Zahra Kargar:** Writing – original draft, Software, Resources, Methodology, Data curation. **Abdollah Hematian Sourki:** Writing – review & editing, Writing – original draft, Visualization, Validation, Supervision, Software, Resources, Project administration, Methodology, Investigation, Formal analysis, Data curation, Conceptualization.

## Declaration of competing interest

The authors declare that they have no known competing financial interests or personal relationships that could have appeared to influence the work reported in this paper.

## Data Availability

The datasets used and/or analyzed during the current study are available from the corresponding author on reasonable request.
